# 2-Methyl-3-(4-nitro­phen­yl)acrylic acid

**DOI:** 10.1107/S1600536808024999

**Published:** 2008-08-06

**Authors:** Niaz Muhammad, M. Nawaz Tahir, Zia-ur-Rehman, Saqib Ali

**Affiliations:** a Department of Chemistry, Quaid-i-Azam University, Islamabad 45320, Pakistan; b University of Sargodha, Department of Physics, Sargodha, Pakistan

## Abstract

The title compound, C_10_H_9_NO_4_, forms *R*_2_^2^(8) dimers due to inter­molecular O—H⋯O hydrogen bonding in the crystal structure. Two dimers are further linked to each other through two inter­molecular C—H⋯O hydrogen bonds, forming an *R*_3_^3^(7) ring motif. The nitro groups form an intra­molecular C—H⋯O hydrogen bond mimicking a five-membered ring. As a result of these hydrogen bonds, polymeric sheets are formed. The aromatic ring makes a dihedral angle of 42.84 (8)° with the carboxyl­ate group and an angle of 8.01 (14)° with the nitro group. There is a π-inter­action (N—O⋯π) between the nitro group and the aromatic ring, with a distance of 3.7572 (14) Å between the N atom and the centroid of the aromatic ring.

## Related literature

For related literature, see: Bernstein *et al.* (1995 ▶); Fujii *et al.* (2002 ▶); Ma & Hayes (2004 ▶); Muhammad *et al.* (2007 ▶, 2008*a* ▶,*b* ▶); Muhammad, Ali, Tahir & Zia-ur-Rehman (2008 ▶); Muhammad, Tahir, Ali, Zia-ur-Rehman & Kashmiri (2008 ▶); Muhammad, Tahir, Zia-ur-Rehman & Ali (2008 ▶); Muhammad, Tahir, Zia-ur-Rehman, Ali & Shaheen, 2008 ▶); Niaz *et al.* (2008 ▶).
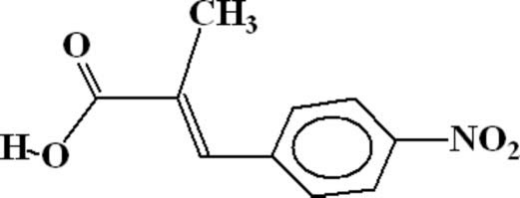


## Experimental

### 

#### Crystal data

C_10_H_9_NO_4_
*M* *_r_* = 207.18
Triclinic, 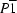

*a* = 7.3878 (3) Å
*b* = 8.1050 (5) Å
*c* = 8.3402 (4) Å
α = 75.793 (2)°
β = 81.835 (3)°
γ = 87.686 (2)°
*V* = 479.21 (4) Å^3^
*Z* = 2
Mo *K*α radiation
μ = 0.11 mm^−1^
*T* = 296 (2) K
0.25 × 0.20 × 0.18 mm

#### Data collection

Bruker Kappa APEXII CCD diffractometer
Absorption correction: multi-scan (*SADABS*; Bruker, 2005 ▶) *T*_min_ = 0.970, *T*_max_ = 0.981
9039 measured reflections
2518 independent reflections
1926 reflections with *I* > 2σ(*I*)
*R*_int_ = 0.023

#### Refinement

*R*[*F* ^2^ > 2σ(*F* ^2^)] = 0.042
*wR*(*F* ^2^) = 0.134
*S* = 1.02
2518 reflections
140 parameters
H atoms treated by a mixture of independent and constrained refinement
Δρ_max_ = 0.24 e Å^−3^
Δρ_min_ = −0.21 e Å^−3^

### 

Data collection: *APEX2* (Bruker, 2007 ▶); cell refinement: *APEX2*; data reduction: *SAINT* (Bruker, 2007 ▶); program(s) used to solve structure: *SHELXS97* (Sheldrick, 2008 ▶); program(s) used to refine structure: *SHELXL97* (Sheldrick, 2008 ▶); molecular graphics: *ORTEP-3 for Windows* (Farrugia, 1997 ▶) and *PLATON* (Spek, 2003 ▶); software used to prepare material for publication: *WinGX* (Farrugia, 1999 ▶) and *PLATON*.

## Supplementary Material

Crystal structure: contains datablocks global, I. DOI: 10.1107/S1600536808024999/cs2087sup1.cif

Structure factors: contains datablocks I. DOI: 10.1107/S1600536808024999/cs2087Isup2.hkl

Additional supplementary materials:  crystallographic information; 3D view; checkCIF report

## Figures and Tables

**Table 1:** Hydrogen-bond geometry (Å, °)

*D*—H⋯*A*	*D*—H	H⋯*A*	*D*⋯*A*	*D*—H⋯*A*
O1—H1⋯O2^i^	0.93 (2)	1.71 (2)	2.6333 (15)	177 (2)
C3—H3⋯O1	0.93	2.31	2.7080 (17)	105
C8—H8⋯O1^ii^	0.93	2.55	3.3471 (17)	144
C9—H9⋯O2^iii^	0.93	2.60	3.4912 (17)	161

Symmetry codes: (i) 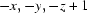; (ii) 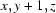; (iii) 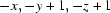.

**Fig. 1.**
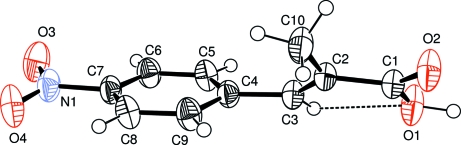
ORTEP drawing of the title compound, C11H12O2 with the atom numbering scheme. The thermal ellipsoids are drawn at the 50% probability level. H-atoms are shown by small circles of arbitrary radii. The intramolecular H-bonds are shown by dotted lines.

**Fig. 2.**
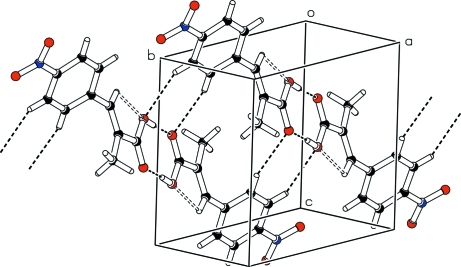
The packing figure (PLATON: Spek, 2003) which shows the dimeric nature of the compound and the interlinkages of the dimers.

## supplementary crystallographic information

### Comment

Cinnamic acid derivatives are widely used chemicals in a variety of fields (Ma *et al.*, 2004). They have been applied as antibacterial agents for suppression of bacterial growth (Fujii *et al.*, 2002). In wine, cinnamic acid and its derivatives join benzoic acid derivatives and flavonoids in creating pigments and tannin agents that give each vintage its characteristic bouquet and color. The title compound has been prepared in continuation of synthesizing various derivatives of cinamic acids (Niaz *et al.*, 2008; Muhammad, Ali, Tahir & Zia-ur-Rehman, 2008; Muhammad, Tahir, Ali, Zia-ur-Rehman & Kashmiri, 2008) and their tin complexes (Muhammad *et al.*, 2008*a*,*b*).

The crystal structures of 3-(4-isopropylphenyl)-2-methylacrylic acid (Muhammad, Tahir, Ali, Zia-ur-Rehman & Kashmiri, 2008), of 3-(4-clorophenyl)-2-methylacrylic acid (Muhammad, Tahir, Zia-ur-Rehman, Ali & Shaheen, 2008) and of 3-(4-bromophenyl)-2-methylacrylic acid (Muhammad *et al.*, 2007) have been reported. The title compound differs from these compounds due to the nitro group at *para* position. In the crystal structure of the title compound, the exocyclic C_sp2_—C_sp2_ bonds are of 1.4770 (18) and 1.4880 (18) Å, the C==C is of 1.3376 (18) Å. The C—O bond length 1.2996 (16) Å is normal, much like the C=O bond length of 1.2300 (15) Å. The resonant N—O bond lengths are equal (1.2185 (16) and 1.2204 (17) Å). There is an interamolecular H-bond of C—H···O type (Table 1, Fig 1). Centrosymmetric *R*_2_^2^(8) dimers (Bernstein *et al.* 1995) are formed due to the intermolecular O1—H1···O2^i^ [symmetry code: i = -*x*, -*y*, -*z* + 1] hydrogen bonding. Two adjacent dimers are linked to each other through two intermolecular H-bonds of C—H···O type forming an *R*_3_^3^(7) motif (Bernstein *et al.* 1995). The group of two dimers are linked to each other by intermolcular H-bonding (Table 1, Fig 2). There exist an N1—O4···*Cg*^ii^ [symmetry code: ii = -*x* + 1, -*y* + 2, -*z*] interaction with a distance of 3.7572 (14) Å between the N-atom and the centroid of the (C4—C9) aromatic ring. The aromatic ring makes a dihedral angle of 42.84 (8)° with with the carboxylate (O1/C1/O2) moiety and 8.01 (14)° with the (N1/O3/O4) nitro group. Due to the intermolecular H-bonding polymeric sheets are formed.

### Experimental

The title compound was prepared according to a reported procedure (Muhammad *et al.*, 2007). A mixture of 4-nitrobenzaldehyde (1.51 g, 10 mmol), methylmalonic acid (2.36 g, 20 mmol) and piperidine (1.98 ml, 20 mmol) in a pyridine (12.5 ml) solution was heated on a steam-bath for 24 h. The reaction mixture was cooled and added to a mixture of 25 ml of concentrated HCl and 50 g of ice. The precipitate formed in the acidified mixture was filtered off and washed with ice-cold water. The product was recrystallized from ethanol. The yield was 79%.

### Refinement

The coordinates of H-atom attached with O1 were refined. The H-atoms attached with C-atoms were positioned geometrically, C—H = 0.93, and 0.96 Å for aromatic and methyl H, and constrained to ride on their parent atoms. The H-atoms were treated as isotropic with *U*_iso_(H) = *xU*_eq_(C,*O*), where *x* = 1.5 for methyl H, and *x* = 1.2 for all other H atoms.

### Crystal data

**Table:**

C_10_H_9_NO_4_	*Z* = 2
*M**_r_* = 207.18	*F*_000_ = 216
Triclinic, *P*1	*D*_x_ = 1.436 Mg m^−^^3^
Hall symbol: -P 1	Mo *K*α radiation λ = 0.71073 Å
*a* = 7.3878 (3) Å	Cell parameters from 2518 reflections
*b* = 8.1050 (5) Å	θ = 2.5–29.1º
*c* = 8.3402 (4) Å	µ = 0.11 mm^−^^1^
α = 75.793 (2)º	*T* = 296 (2) K
β = 81.835 (3)º	Prismatic, colourless
γ = 87.686 (2)º	0.25 × 0.20 × 0.18 mm
*V* = 479.21 (4) Å^3^	

### Data collection

**Table:**

Bruker Kappa APEXII CCD diffractometer	2518 independent reflections
Radiation source: fine-focus sealed tube	1926 reflections with *I* > 2σ(*I*)
Monochromator: graphite	*R*_int_ = 0.023
Detector resolution: 7.4 pixels mm^-1^	θ_max_ = 29.1º
*T* = 296(2) K	θ_min_ = 2.5º
ω scans	*h* = −10→9
Absorption correction: multi-scan(SADABS; Bruker, 2005)	*k* = −11→9
*T*_min_ = 0.970, *T*_max_ = 0.981	*l* = −11→11
9039 measured reflections	

### Refinement

**Table:**

Refinement on *F*^2^	Secondary atom site location: difference Fourier map
Least-squares matrix: full	Hydrogen site location: inferred from neighbouring sites
*R*[*F*^2^ > 2σ(*F*^2^)] = 0.042	H atoms treated by a mixture of independent and constrained refinement
*wR*(*F*^2^) = 0.134	*w* = 1/[σ^2^(*F*_o_^2^) + (0.0713*P*)^2^ + 0.0915*P*] where *P* = (*F*_o_^2^ + 2*F*_c_^2^)/3
*S* = 1.02	(Δ/σ)_max_ < 0.001
2518 reflections	Δρ_max_ = 0.24 e Å^−^^3^
140 parameters	Δρ_min_ = −0.21 e Å^−^^3^
Primary atom site location: structure-invariant direct methods	Extinction coefficient: ?

### Fractional atomic coordinates and isotropic or equivalent isotropic displacement parameters (Å^2^)

**Table:**

	*x*	*y*	*z*	*U*_iso_*/*U*_eq_	
O1	0.04661 (16)	0.13519 (13)	0.29456 (12)	0.0547 (4)	
O2	0.09796 (15)	0.16087 (12)	0.54300 (12)	0.0526 (3)	
O3	0.52309 (18)	1.11600 (15)	−0.36244 (14)	0.0661 (4)	
O4	0.35761 (19)	1.23296 (13)	−0.18699 (15)	0.0648 (4)	
N1	0.41247 (18)	1.10856 (14)	−0.23745 (14)	0.0466 (4)	
C1	0.10453 (16)	0.21896 (15)	0.39150 (15)	0.0357 (3)	
C2	0.17797 (17)	0.39231 (15)	0.31101 (16)	0.0362 (3)	
C3	0.17195 (18)	0.45348 (15)	0.14759 (16)	0.0378 (3)	
C4	0.22936 (17)	0.62471 (15)	0.04743 (15)	0.0361 (3)	
C5	0.3308 (2)	0.64219 (16)	−0.11015 (16)	0.0424 (4)	
C6	0.39099 (19)	0.80039 (17)	−0.20464 (16)	0.0425 (4)	
C7	0.34487 (18)	0.94026 (15)	−0.14089 (15)	0.0375 (4)	
C8	0.2375 (2)	0.92912 (16)	0.01030 (17)	0.0432 (4)	
C9	0.1804 (2)	0.76957 (16)	0.10462 (16)	0.0430 (4)	
C10	0.2580 (2)	0.47901 (17)	0.42356 (17)	0.0480 (4)	
H1	−0.005 (3)	0.032 (3)	0.355 (2)	0.0656*	
H3	0.12727	0.38114	0.09157	0.0454*	
H5	0.35821	0.54652	−0.15208	0.0508*	
H6	0.46089	0.81202	−0.30868	0.0510*	
H8	0.20402	1.02624	0.04821	0.0518*	
H9	0.10844	0.75935	0.20750	0.0516*	
H10A	0.33667	0.40104	0.48836	0.0719*	
H10B	0.16126	0.51574	0.49695	0.0719*	
H10C	0.32723	0.57588	0.35757	0.0719*	

### Atomic displacement parameters (Å^2^)

**Table:**

	*U*^11^	*U*^22^	*U*^33^	*U*^12^	*U*^13^	*U*^23^
O1	0.0855 (8)	0.0371 (5)	0.0397 (5)	−0.0259 (5)	−0.0071 (5)	−0.0026 (4)
O2	0.0801 (7)	0.0381 (5)	0.0357 (5)	−0.0229 (5)	−0.0058 (5)	0.0008 (4)
O3	0.0862 (8)	0.0524 (7)	0.0487 (6)	−0.0249 (6)	0.0068 (6)	0.0033 (5)
O4	0.1010 (9)	0.0290 (5)	0.0614 (7)	−0.0085 (5)	−0.0120 (6)	−0.0036 (5)
N1	0.0638 (7)	0.0340 (6)	0.0385 (6)	−0.0119 (5)	−0.0133 (5)	0.0030 (5)
C1	0.0399 (6)	0.0288 (6)	0.0353 (6)	−0.0057 (5)	−0.0021 (5)	−0.0028 (4)
C2	0.0379 (6)	0.0278 (5)	0.0392 (6)	−0.0052 (5)	−0.0024 (5)	−0.0019 (5)
C3	0.0443 (6)	0.0284 (6)	0.0380 (6)	−0.0068 (5)	−0.0037 (5)	−0.0029 (5)
C4	0.0416 (6)	0.0294 (6)	0.0342 (6)	−0.0041 (5)	−0.0064 (5)	−0.0004 (4)
C5	0.0569 (8)	0.0300 (6)	0.0375 (6)	−0.0005 (5)	−0.0009 (5)	−0.0062 (5)
C6	0.0521 (7)	0.0360 (6)	0.0338 (6)	−0.0020 (5)	0.0023 (5)	−0.0023 (5)
C7	0.0468 (7)	0.0281 (6)	0.0343 (6)	−0.0062 (5)	−0.0093 (5)	0.0017 (5)
C8	0.0607 (8)	0.0292 (6)	0.0380 (6)	0.0016 (5)	−0.0052 (6)	−0.0062 (5)
C9	0.0554 (8)	0.0348 (6)	0.0334 (6)	−0.0009 (5)	0.0030 (5)	−0.0029 (5)
C10	0.0626 (8)	0.0353 (7)	0.0437 (7)	−0.0160 (6)	−0.0124 (6)	0.0001 (5)

### Geometric parameters (Å, °)

**Table:**

O1—C1	1.2996 (16)	C5—C6	1.3832 (19)
O2—C1	1.2300 (15)	C6—C7	1.3774 (19)
O3—N1	1.2204 (17)	C7—C8	1.3759 (19)
O4—N1	1.2185 (16)	C8—C9	1.3855 (19)
O1—H1	0.93 (2)	C3—H3	0.9300
N1—C7	1.4698 (17)	C5—H5	0.9300
C1—C2	1.4880 (18)	C6—H6	0.9300
C2—C3	1.3376 (18)	C8—H8	0.9300
C2—C10	1.4965 (19)	C9—H9	0.9300
C3—C4	1.4770 (18)	C10—H10A	0.9600
C4—C9	1.3887 (18)	C10—H10B	0.9600
C4—C5	1.3951 (18)	C10—H10C	0.9600
			
O1···C8^i^	3.3471 (17)	C4···H10C	2.7200
O1···C6^ii^	3.4128 (19)	C4···H3^ii^	3.0300
O1···O2^iii^	2.6333 (15)	C9···H10C	2.6400
O2···O1^iii^	2.6333 (15)	C10···H9	2.8300
O2···C1^iii^	3.3657 (16)	C10···H10B^v^	3.0700
O2···N1^iv^	3.1112 (17)	H1···O1^iii^	2.882 (17)
O1···H1^iii^	2.882 (17)	H1···O2^iii^	1.71 (2)
O1···H3	2.3100	H1···C1^iii^	2.59 (2)
O1···H8^i^	2.5500	H1···H1^iii^	2.36 (2)
O2···H10B	2.8600	H3···O1	2.3100
O2···H10A	2.5900	H3···H5	2.5900
O2···H1^iii^	1.71 (2)	H3···C4^ii^	3.0300
O2···H9^v^	2.6000	H5···O4^i^	2.6300
O3···H6	2.4400	H5···H3	2.5900
O3···H10A^vi^	2.7600	H6···O3	2.4400
O3···H6^vii^	2.6500	H6···O3^vii^	2.6500
O3···H10C^viii^	2.7800	H6···C2^x^	3.0800
O4···H5^ix^	2.6300	H8···O1^ix^	2.5500
O4···H8	2.4200	H8···O4	2.4200
O4···H10A^vi^	2.7400	H9···C2	2.9300
O4···H10C^viii^	2.8500	H9···C10	2.8300
N1···O2^vi^	3.1112 (17)	H9···H10C	2.4200
N1···C8^viii^	3.378 (2)	H9···O2^v^	2.6000
C1···O2^iii^	3.3657 (16)	H10A···O2	2.5900
C2···C6^x^	3.5837 (19)	H10A···O3^iv^	2.7600
C6···O1^ii^	3.4128 (19)	H10A···O4^iv^	2.7400
C6···C2^x^	3.5837 (19)	H10B···O2	2.8600
C8···N1^viii^	3.378 (2)	H10B···C1^v^	3.0800
C8···O1^ix^	3.3471 (17)	H10B···C2^v^	2.9500
C9···C10	3.1937 (19)	H10B···C10^v^	3.0700
C10···C9	3.1937 (19)	H10B···H10B^v^	2.4000
C1···H10B^v^	3.0800	H10C···C4	2.7200
C1···H1^iii^	2.59 (2)	H10C···C9	2.6400
C2···H9	2.9300	H10C···H9	2.4200
C2···H10B^v^	2.9500	H10C···O3^viii^	2.7800
C2···H6^x^	3.0800	H10C···O4^viii^	2.8500
			
C1—O1—H1	111.6 (12)	C7—C8—C9	118.33 (12)
O3—N1—O4	123.45 (13)	C4—C9—C8	120.81 (12)
O3—N1—C7	118.21 (12)	C2—C3—H3	117.00
O4—N1—C7	118.33 (12)	C4—C3—H3	117.00
O1—C1—O2	122.55 (12)	C4—C5—H5	120.00
O1—C1—C2	116.77 (11)	C6—C5—H5	120.00
O2—C1—C2	120.68 (11)	C5—C6—H6	121.00
C1—C2—C10	115.28 (11)	C7—C6—H6	121.00
C3—C2—C10	126.40 (12)	C7—C8—H8	121.00
C1—C2—C3	118.29 (11)	C9—C8—H8	121.00
C2—C3—C4	126.35 (12)	C4—C9—H9	120.00
C3—C4—C9	121.47 (11)	C8—C9—H9	120.00
C5—C4—C9	119.09 (12)	C2—C10—H10A	109.00
C3—C4—C5	119.41 (11)	C2—C10—H10B	109.00
C4—C5—C6	120.66 (12)	C2—C10—H10C	109.00
C5—C6—C7	118.38 (12)	H10A—C10—H10B	109.00
N1—C7—C6	119.04 (11)	H10A—C10—H10C	110.00
N1—C7—C8	118.35 (11)	H10B—C10—H10C	109.00
C6—C7—C8	122.61 (12)		
			
O3—N1—C7—C6	−7.7 (2)	C2—C3—C4—C9	−44.9 (2)
O3—N1—C7—C8	172.34 (13)	C3—C4—C5—C6	−178.09 (13)
O4—N1—C7—C6	173.58 (14)	C9—C4—C5—C6	3.7 (2)
O4—N1—C7—C8	−6.4 (2)	C3—C4—C9—C8	179.06 (13)
O1—C1—C2—C3	3.07 (18)	C5—C4—C9—C8	−2.7 (2)
O1—C1—C2—C10	−174.99 (12)	C4—C5—C6—C7	−1.4 (2)
O2—C1—C2—C3	−176.04 (13)	C5—C6—C7—N1	178.15 (13)
O2—C1—C2—C10	5.90 (18)	C5—C6—C7—C8	−1.9 (2)
C1—C2—C3—C4	176.84 (12)	N1—C7—C8—C9	−177.24 (13)
C10—C2—C3—C4	−5.3 (2)	C6—C7—C8—C9	2.8 (2)
C2—C3—C4—C5	136.88 (15)	C7—C8—C9—C4	−0.4 (2)

Symmetry codes: (i) *x*, *y*−1, *z*; (ii) −*x*, −*y*+1, −*z*; (iii) −*x*, −*y*, −*z*+1; (iv) *x*, *y*−1, *z*+1; (v) −*x*, −*y*+1, −*z*+1; (vi) *x*, *y*+1, *z*−1; (vii) −*x*+1, −*y*+2, −*z*−1; (viii) −*x*+1, −*y*+2, −*z*; (ix) *x*, *y*+1, *z*; (x) −*x*+1, −*y*+1, −*z*.

### Hydrogen-bond geometry (Å, °)

**Table:**

*D*—H···*A*	*D*—H	H···*A*	*D*···*A*	*D*—H···*A*
O1—H1···O2^iii^	0.93 (2)	1.71 (2)	2.6333 (15)	177 (2)
C3—H3···O1	0.93	2.31	2.7080 (17)	105
C8—H8···O1^ix^	0.93	2.55	3.3471 (17)	144
C9—H9···O2^v^	0.93	2.60	3.4912 (17)	161

Symmetry codes: (iii) −*x*, −*y*, −*z*+1; (ix) *x*, *y*+1, *z*; (v) −*x*, −*y*+1, −*z*+1.
